# Supplementary material for: One mother for two species via obligate cross-species cloning in ants
Source: Nature. 2025 Sep 3;646(8084):372–7. doi: 10.1038/s41586-025-09425-w (PMC12507663; doi:10.1038/s41586-025-09425-w)
Supplement: Supplementary file 1 — This file includes Supplementary Notes 1–4, Figs. 1–11 and Tables 2–4, which support the conclusions of the main article, and references. [file 41586_2025_9425_MOESM1_ESM.pdf]

nature portfolio

https://doi.org/10.1038/s41586-025-09425-w

Supplementary information

One mother for two species via obligate cross-species cloning in ants

In the format provided by the authors and unedited

Supplementary Information for

One mother for two species: obligate cross-species cloning in ants

Y. Juvé[1]†, C. Lutrat[1]†, A. Ha†[1], A. Weyna[1,2], E. Lauroua[1], A. C. Afonso Silva[3], C. Roux[3], E. Schifani[4,5], C. Galkowski[6], C. Lebas[7], R. Allio[8], I. Stoyanov[9], N. Galtier[1], B.C. Schlick-Steiner[10], F.M. Steiner[10], D. Baas[11], B. Kaufmann[12], J. Romiguier[1]*

Affiliations:

[1]ISEM - Univ Montpellier, CNRS, IRD; Montpellier, F-34095, France

[2]Department of Ecology and Evolution, Univ Lausanne; Lausanne, Suisse

[3]Univ. Lille, CNRS, UMR 8198 - Evo-Eco-Paleo, F-59000, Lille, France

[4]Department of Chemistry, Life Sciences, and Environmental Sustainability, University of Parma; Parma, 43124, Italy

[5]Institut de Biologia Evolutiva (CSIC-Univ. Pompeu Fabra); Barcelona, 08003, Spain

[6]Antarea, 104 route de Mounic; Saint-Aubin-de-Médoc, F-33160, France

[7]2 impasse del Ribas; Canohes, F-66680, France

[8]Centre de Biologie pour la Gestion des Populations, INRAE, CIRAD, IRD, Montpellier SupAgro, Université de Montpellier; Montpellier, F-34988, France

[9]Department of Developmental Biology, Faculty of Biology, University of Plovdiv "Paisii Hilendarski", Faculty of Biology, 24, Tzar Assen Str., Plovdiv, Bulgaria

[10]Department of Ecology, Universität Innsbruck; Innsbruck, 6020, Austria

[11]Institut NeuroMyoGène, CNRS UMR-5284, INSERM U-1314, MeLis, Université Lyon, Université Claude Bernard Lyon 1, F-69008 Lyon, France

[12]Université Claude Bernard Lyon 1, LEHNA UMR 5023, CNRS, ENTPE, F-69622, Villeurbanne, France

† These authors contributed equally to this work.

* Corresponding author. Email: jonathan.romiguier@umontpellier.fr

This PDF file includes:

Supplementary Note 1 to 3
Supplementary Figures 1 to 11
Supplementary Tables 2 to 4
SI References

Other supporting materials for this manuscript include the following:

Supplementary Tables 1 and 5 (.xls)

SI Guide

Supplementary Notes..3
Supplementary Note 1: Species identifications on *M. ibericus* brood confirm that queens regularly lay *M. structor* males..3
Supplementary Note 2: Worker reproduction and hybrid queen in artificial conditions..4
Supplementary Note 3: Description of *Messor structor* clonal males (clonal morph)...5
Supplementary Note 4: Historical hybridization and species delimitation of the clonal male lineage..................8

Supplementary Figures..14
Supplementary Fig. 1..14
Supplementary Fig. 2..15
Supplementary Fig. 3..16
Supplementary Fig. 4..17
Supplementary Fig. 5..18
Supplementary Fig. 6..19
Supplementary Fig. 7..20
Supplementary Fig. 8..21
Supplementary Fig. 9..22
Supplementary Fig. 10..23
Supplementary Fig. 11..24

Supplementary Tables..25
Supplementary Table 1..25
Supplementary Table 2..26
Supplementary Table 3..27
Supplementary Table 4..28
Supplementary Table 5..29

Supplementary Notes

Supplementary Note 1: Species identifications on *M. ibericus* brood confirm that queens regularly lay *M. structor* males

To check the caste/species composition of *M. ibericus* colony production, we collected the brood (eggs + larvae) of 5 *M. ibericus* lab colonies and placed them in a dilution buffer before performing a total of 428 DNA extractions. We then conducted PCR tests for species identification for each of them. Reliable amplifications of 286 out of the 428 DNA extracts allowed to identify caste and species, with a total of 194 hybrid workers, 59 *M. ibericus* (queens or males) and 33 *M. structor* males (see Supplementary Table 2 for details, Supplementary Fig. 2 for the agarose gel electrophoresis results).

To check whether these *M. structor* males were laid by queens and not hybrid workers, we conducted a supplementary analysis. We isolated 16 queens from 7 colonies in individual tubes. We let them lay in the dark at 27 °C with food (dandelion seeds) and collected eggs three times every 48H for a total of 133 eggs. Following the same procedure as the previous experiment, we conducted DNA extractions and PCR tests for species identification. Amplification of 78 DNA extracts allowed to identify 65 hybrid workers, 6 *M. ibericus* males/queens and 7 *M. structor* males (see Supplementary Table 3 for details, Supplementary Fig. 3 for the agarose gel electrophoresis results).

Collectively, these results confirm that queens regularly lay eggs of *M. structor* (total of 11% of identified laid eggs). Such a high proportion contrasted with the rarity of *M. structor* clonal males that developed as adults in the lab from our *M. ibericus* colonies (only 2 observations from 65 colonies monitored from 12 to 24 months). This suggests that *M. ibericus* queens or workers actively manage the survival of *M. structor* clonal males and select whether corresponding eggs/larvae are allowed to reach adulthood or not.

Supplementary Note 2: Worker reproduction and hybrid queen in artificial conditions

In a closely related species of *M. ibericus, M. barbarus* workers from queenless colonies are able to lay eggs that develop into males. Because workers are all hybrids between two genetic lineages, these males harbor mosaic haploid genomes that are a mix of alleles from the two genetic lineages, as expected in case under worker parthenogenesis[12]. We sequenced the genome of 3 males from queenless lab colonies of *M. ibericus* and confirmed that the same phenomenon can occur. These male genomes feature a very low genetic diversity (average of 0.027) but a mix of *M. structor* and *M. ibericus* alleles (see Supplementary Table 1 for individuals SH13-05, SH13-11, SH18-20), which indicates that they are haploid mosaics with loci that are either specific to one or the other species. We found no such mosaic males from field collected individuals, which indicates that they are rarely produced or not viable in natural conditions. This mirrors the observations in some other genetic caste determining species such as *Messor barbarus*[12], *Anoplolepis gracilipes*[12,19] and *Pogonomyrmex* lineages[89].

Rare occurrences of hybrid queens can occur in *Pogonomyrmex* or *Anoplolepis gracilipes* with genetic caste determination[19,90]. We did not find such examples in natural populations of *M. ibericus*, but obtained such a virgin queen from a laboratory colony (ORT3P2 in Supplementary Table 1). As in other species, the rarity of such cases and the strong genetic divergence between lineages indicates that these queens either do not occur in natural conditions or have a sufficiently low fertility to not contribute to significant gene flow.

Supplementary Note 3: Description of *Messor structor* clonal males (clonal morph)

Messor structor clonal males (Fig. 3e) exhibit important morphological and genetic differences compared with other male relatives (*M. ibericus* nestmates and *M. structor* "wild-type" morph, Fig. 2a and Fig. 3d, respectively). We identified at least five morphological distinctive criteria that are detailed in the formal description below and in Supplementary Table 5. Technical morphological terms are illustrated in Supplementary Fig. 4 for help. Distinctive criteria and comparisons between different males are illustrated in Supplementary Fig. 5. Principal component analysis and plot illustrating hair density variations are available in Supplementary Fig. 6. Full pictures of the three males side by side are available in Extended Data Fig. 6, along with pictures of *M. ibericus* queen and worker.

We detail the morphological specificities of the clonal morph based on 9 individuals compared with 7 individuals each of males from *M. ibericus* and *M. structor* "wild-type" lineage (Supplementary Table 5).

***Messor structor* (clonal morph)**
(Fig. 2b, Fig. 3e, Extended Data Fig. 4 and 6, Supplementary Fig. 4, Supplementary Fig. 6, Supplementary Table 4, Supplementary Table 5)
Type material as designated hereby: *Holotype*. FRANCE MTNm15 | Estagel | 23/03/2022 | N 42.7619592° E 2.6995636° | Y. Juvé | Holotypus | "*Messor*" | *"structor"* (stored at "Institut des Sciences de l'Evolution de Montpellier" Collection)
Paratypes: 6 males (see Supplementary Table 5).
Other material examined: *M. structor* (wild-type) and *M. ibericus* males (see Supplementary Table 5)

Diagnosis. Easily distinguishable from *M. ibericus* and *M. structor* males. Generally, slightly larger than other males. Pilosity less abundant than in the other two males and particularly reduced and heterogeneous on the mesosoma (mesoscutum; Supplementary Figs. 5 and 6b) or the anterior half of the first gastral sternite (particularly useful to differentiate *M. ibericus* and *M. structor* males, with high and low hair density, respectively, Supplementary Figs. 5 and 6b). Mesoscutum and mesoscutellum higher and more globular than in the two other males. The apical angle of the petiole is characteristically obtuse (>100°) compared with *M. structor* (>90°) and *M. ibericus* (<90°) (Supplementary Fig. 5 and 6b). Pterostigmas characteristically nucleated in brown with lighter peripheral banding (Supplementary Fig. 5). Sculptures less smooth and shiny than in other males. Observation of the anepisternum is a good discriminant of *M. ibericus*, as the cuticle is matte and evenly microreticulated, whereas it is shiny and smoother in the others (Supplementary Fig. 5). This criterion is similar to the main distinctive criterion of *M. ibericus* queens[13]. *M. structor* wild males seem to be the most morphologically diverse (Supplementary Fig. 6), which is consistent with their higher genetic diversity (Supplementary Table 4).

Five morphological criteria (detailed in Supplementary Table S5 and Supplementary Fig. 5) discriminate them very well from *M. structor* and *M. ibericus* males, forming the most divergent group in a principal component analysis (Supplementary Fig. 6a).

Description. <u>Head</u>: Uniformly black. Apex of mandibles brown. Torulus, antennal socket and junction of flagellomeres yellowish. Face longitudinally striated. Striae closest to the eye curve in an arch behind it, but tend to fade or to be intermittent towards the temple. Faint microreticulations on temples and lateral parts of the occiput. Mandible with 9 teeth, sometimes

with one or two more smaller denticles. Palp formula 4:3. Clypeus convex with its anterior margin slightly depressed. Glabrous compound eyes with at least 400 ommatidia. Scape short, not reaching occipital margin, with its base flared. Funicle with twelve articles. Pedicel wider than the other flagellomeres. Second article longer than the following one. Entire antenna covered with yellowish bristles. Some are longer than others on the scape and the pedicel. Other articles covered with dense, uniform pubescence. Viewed from the side, face is crowned with more or less long setae that converge towards the forehead. Other groups of erect, size-variable setae are visible on the clypeus and the gula, but less marked than on the face. Gena almost glabrous, at least in the area directly under the eye. Mesosoma: Uniformly black. Legs lighten distally, down to the tarsi, which are yellow. Wings and pilosity yellowish. Mesoscutum rounded and shiny. Microreticulation very weak or absent. A few striae along the side, but smooth overall. Setosity heterogeneous and weak. Some setae are long, acuminate and sporadic, while the majority, located around the anterior border, are short and blunt, as if truncated. Scutoscutellar sulcus deeply impressed and crossed by cuticular folds forming cells. Mesoscutellum globose. Striation runs from interalar base of the sulcus, arching behind the hindwing. Top of mesoscutellum rougher than mesoscutum.Wings slightly smoky, yellowed. Pale yellow venation. Depending on the angle of the light source, wingsurfaces may appear iridescent. Posterior margins showing pubescence. Pterostigmas nucleated in brown with paler and translucent peripheral venation. Submarginal cell 2 sometimes split in two at its first proximal third. Other specimens show an incomplete separation or a fully unconstricted cell. The Rs + M vein (radial sector + media), which joins the discoidal cell to the submarginal cell, may be of differing length or even non-existent in cases where the two cells touch. There may also be a short perpendicular vein running from the middle of the 2rs + m vein (radial sector-medial cross vein). These variations can be observed between the two forewings of the same specimen. Pronotum with microreticulations and lateral striations. Anepisternum less microreticulated and striated, especially near the center where sculpture is absent thus allowing the cuticle to shine. Mesopleural sulcus has the same appearance as the scutoscutellar sulcus. It runs around the anepisternum as far as the subalar area where cuticular folds form elongated cells. Katepisternum has lateral striae which also tend to fade or be intermittent towards the center. Microreticulations weak. A cluster of bristles visible under the mesopectum. Metapleuron laterally striated and propodeum longitudinally striated. Microreticulations deepening towards the posterior part. Group of straight setae on the sloping side of the propodeum, which is slightly humped. Propodeal spiracle semicircular, facing backwards due to its position on a globular protuberance of the cuticle.Ventral side of coxae hairy, rather dark. Trochanters lighter than coxae and femurs. Femurs smooth and hairy. Pubescence decumbent (10-15°) on upper side. Sub-erect to erect setae (35-45+°) on the underside and lateral parts. Tibiae more densely and evenly pubescent. Tarsi with even finer pubescence, making them yellow. Nodes and gaster: Petiole long and low, pyramidal from apex, and with an angle > 100°. Anterior face slightly concave and pubescent. Posterior face short and straight or very slightly depressed. A few scattered setae on upper parts. Postpetiole rounded with scattered setae, also on its ventral side. Overall, these black nodes are finely microreticulated and their upper faces are slightly rough. Gaster mainly black. Sclerite posterior margins brown-yellow. Overall smooth and shiny. A few faint cuticle folds on the flanks of the first tergite. The latter is covered with short setae on the top. First sternite much less so, sometimes completely hairless. Posterior margin of other sclerites with several rows of setae, longer towards the center than on the lateral edges. Genitalia more or less light brown, with occasional shaded areas.

Nuclear DNA: Distinct from their *M. structor* “wild” counterparts (~2.75 Myrs of divergence; Fig 3c, Extended Data Figs. 1 and 3-4) and *M. ibericus* (~6 Myrs of divergence; Fig 3c, Extended Data Figs. 1 and 3-4). Extremely low genetic diversity and high genetic load (Supplementary Table 4). Sequence alignments available in the zenodo repository (https://zenodo.org/records/11506545.) with following sequence IDs in figS1.fas and figS3.fas:

RFOU2M, SH02-24, SH04-01, SH04-02, SH04-18, SH04-19, SH04-20, SH13-13, SH13-14, SH13-15, SH13-19, SH13-21, SH13-22, SH13-23, SH14-01, SH14-02, SH14-03, SH14-04, SH14-05, SH14-06, SH14-07, SH18-03, SH19-01, SH19-04.

Mitochondrial DNA: Identical to their *M. ibericus* surrogate mothers (Fig. 2 and Extended Data Fig. 1), distinct to their *M. structor* "wild" counterparts (Extended Data Fig. 1). Sequence alignments available in the zenodo repository (https://zenodo.org/records/11506545) with following sequence IDs in figS2.fas: RFOU2M, SH02-24, SH04-01, SH04-02, SH04-18, SH04-19, SH04-20, SH13-13, SH13-14, SH13-15, SH13-19, SH13-21, SH13-22, SH13-23, SH14-01, SH14-02, SH14-03, SH14-04, SH14-05, SH14-06, SH14-07, SH18-03, SH19-01, SH19-04.

Distribution (Fig. 1b, Supplementary Supplementary Table 1): Exactly the same colonies and the same distribution area as *M. ibericus*. Austria, Bulgaria, Croatia, France, Germany, Greece, Italy, Romania, Slovenia, Spain, Switzerland.

Life history: Exclusively composed of males. Characterized as a "domesticated" lineage/morph of *M. structor*, as individuals are not found within *M. structor* nests but are exclusively born within *M. ibericus* nests. Reproduction occurs clonally through the ova of *M. ibericus* queens. Pre-swarming flight activity has been observed near Rüdesheim by G. Heller (males of *M. structor* clonal morph and males of *M. ibericus* swarming around the entrance of the same *M. ibericus* nests). Swarming flights from a *M. ibericus* colony reported in Montpellier on 20/10/2023 by J. Romiguier (males of *M. structor* clonal morph have been seen flying away together with *M. ibericus* queens).

Supplementary Note 4: Historical hybridization and species delimitation of the clonal male lineage

Clonal males appear as in a domesticated situation where they rely on *M. ibericus* colonies to reproduce clonally. This raises two questions:
1) Can they regularly escape from this situation by reproducing with their “wild” *M. structor* female counterparts?
2) Should we consider clonal males as a *M. structor* lineage or as a distinct, male-only species?
We answer these two questions through the 8 following analyses.

Clonal males currently never or rarely mate with wild-type *M. structor* females

To investigate the existence of hybrids between the clonal and wild-type lineage, we analyzed 45 *M. structor* female genomes. Such an analysis is more challenging than screening for clear interspecific hybrids, as the divergence level of the two potential parents is low (average of 0.0015 among loci) compared to that of *M. ibericus* and *M. structor* (average of 0.0052 among loci). To address this challenge, we enhanced our hybrid detection method[15] by employing an *in silico* hybridization approach to adjust for such low heterozygosity cases. For this, we first merged the haploid genomes of a clonal male (id RFOU2M) and a wild-type male (id SH09-22). Positions identified as heterozygous in one of the haploid genome (*i.e.* SNP call errors) were treated as missing data. The resulting artificial hybrid was then analyzed with all other *M. structor* genomes, with results used as expected values for a clonal/wild-type hybrid. SNP heterozygosity and γ hybrid index for each loci was computed as previously described (Heterozygosity and hybrid detection subsection in Methods).

Results for the 45 *M. structor* genomes and artificial clonal/wild-type genome are presented in Supplementary Fig. 7. For each *M. structor* genome, average heterozygosity among loci (n=5856) is significantly lower (up to 6 times) than the expected value in case of clonal/wild-type hybrid (two-sided Wilcoxon rank sum test, p-value $< 2.2 \times 10^{-16}$, Supplementary Fig. 7a). Similarly, the average γ hybrid index computed on the 832 loci common to all metazoa is significantly lower (up to 72 times) than the expected value in case of clonal/wild-type hybrid (two-sided Wilcoxon rank sum test, p-value $< 2.2 \times 10^{-16}$, Supplementary Fig. 7b). This reinforces the view that clonal males are essentially trapped in a domesticated situation, as it confirms that they rarely reproduce sexually with their *M. structor* female counterparts.

Clonal and wild-type lineages have an intertwined origin

To specifically investigate the divergence history between clonal and wild-type lineages, we increased the number of sites analyzed by focusing on 36 high-quality genomes from our phylogenomic dataset. We retained individuals with highly covered genomes, ensuring an average coverage of 52.3X. For each species, we selected 6 individuals, including 6 from the clonal lineage and 6 from the wild-type lineage. This selection was made to accurately represent the geographical and phylogenetic diversity of each species and lineage (see Supplementary Fig. 8a for the ID of individuals), with *M. wasmanni* used as an outgroup. After cleaning the

resulting alignment using the automated procedure of *trimal*[80], we increased the alignment size from 2,780,573 to 11,102,444 base pairs. We inferred a phylogenetic tree using *IQ-TREE* (v. 2.2.2.7)[79] with a GTR+I+F+G4 model and 1,000 ultrafast bootstrap (-bb 1000). We also estimated Site Concordance Factors (sCF), defined as the percentage of decisive alignment sites supporting a branch in the resulting tree[91]. The topology, bootstrap and sCF support are available in Supplementary Fig. 8a.

While the monophyly of each species was strongly supported by maximal bootstrap values (100), the monophyly of the *M. structor* wild-type lineage received poor support (bootstrap value of 54). This contrasts with our previous phylogenetic analysis (Extended Data Fig. 1), which included all individuals but had a smaller alignment size (2,780,573 bp), where this node was supported by a bootstrap value of 100. The reduced support appears to be due to high conflicting signals for this node when considering a more significant part of the genome (11,102,444 bp). Specifically, less than half of decisive sites supported the monophyly of the two main wild-type lineages (divided into a western and an eastern lineage), as indicated by a Site Concordance Factor of 39%. Conversely, more than half of decisive sites supported the clonal lineage as nested within *M. structor* wild-type individuals. Of these, 32.6% supported western wild-type individuals (Y16627-1, Y15267-1 and Y15268-1) as the closest relatives of the clonal lineage, while 28.6% supported eastern wild-type individuals (Y14750-1, RBUDAW and Y14582-1).

These results indicate that while the monophyly of *M. structor* is strongly supported (bootstrap value of 100), the divergence among its lineages (one clonal and two wild-type) appear intertwined. This phylogenomics approach further supports the idea that clonal males evolved from wild-type *M. structor* ancestors, as their evolutionary origin appears entangled with them.

Common population ancestry for clonal and wild-type lineages

To further explore whether clonal and wild-type lineages belong to the same species, we conducted a population structure analysis aimed at identifying genetically homogeneous groups of individuals[16]. However, these approaches can be unreliable with uneven sampling[92]. To mitigate this, we applied the analysis to the 36 individual dataset composed of 6 individuals per species + 6 clonal males (see Supplementary Fig. 8a). We selected these individuals from the previously analyzed VCF file, using the same variant filtering criteria as for SNP heterozygosity calculations (see Methods, Heterozygosity and hybrid detection subsection). As before, we used *PLINK*[85] (v1.90b6.21) then *fastStructure*[16] (v. 1.0). *Faststructure* requires the user to manually set the K parameter, which determines the number of genetic clusters to group similar individuals. To identify the optimal number of genetic clusters in our dataset, we ran the program with K values ranging from 2 to 8. We then used the *chooseK.py* script, following the fastStructure manual's recommendations, to compare cross-validation errors across the analyses.

Results for the optimal number of genetic clusters (K=5) are available in Supplementary Fig. 8b. Both clonal and wild-type lineages were inferred to belong to the same genetic cluster, with

ancestry proportions exceeding 0.99 for all individuals. Overall, the ancestry proportions were highly homogeneous and consistent with species assignments, with the lowest proportion being 0.90 for one *M. mcarthuri* individual. These patterns remained consistent when analyzing K values set to 6, 7, or 8. In each case, clonal and wild-type individuals consistently grouped within the same *M. structor* genetic cluster, with ancestry proportions above 0.99.

Triplet-based delimitation (*tr2*) supports Clonal and Wild-type lineages as same species

To assess whether the wild-type and clonal lineages should be considered distinct species, we performed species delimitation analyses using *tr2* (Trinomial Distribution of Triplets)[93]. This approach quantifies concordance and discordance among gene trees, selecting the most likely species delimitation based on a probabilistic model of rooted triplets. We analyzed the same dataset as for Supplementary Fig. 8 (6 representative individuals per species), with individual gene trees inferred using *IQ-TREE* (v. 2.07)[79] with a GTR+I+F+G4 substitution model. *M. mcarthuri* was used as the outgroup, resulting in a dataset of 5,654 gene trees.

To test whether clonal and wild-type lineage should be considered as the same species, we tested alternative species assignments by comparing three species delimitation models: a null model considering each individual as a distinct species, Model 1 considering the wild-type and clonal lineage as separate species (*i.e.* 7 species in total) and Model 2 considering the wild-type and clonal lineage as the same species (*i.e.* 6 species in total).

The model with the lowest Bayesian Information Criterion score (*i.e.* best model) was model 3, grouping the clonal and wild-type lineage as a single species (Null model = 308,469.38; Model 1 = 83,037.33; Model 2 = 50,143.05). This supports that the clonal lineage should be considered as part of the *M. structor* species (Supplementary Fig. 8c).

Quartet-based delimitation (*SODA*) support clonal and wild-type lineage as same species

To confirm the previous species delimitation result, we used *SODA* v.1.0.2 (Species Boundary Delimitation using ASTRAL), an alternative to the triplet-based approach of *tr2* based on quartet frequencies from unrooted gene trees[94]. We used the same gene trees as used for *tr2*. Prior to the analysis, each individual was assigned to its putative species, with clonal and wild-type lineages considered as distinct species *a priori*.

When setting the p-value threshold to 0.05, the delimitation approach merged the wild-type and clonal lineages as a single species. This pattern remained consistent when setting the p-value threshold to 0.01 or 0.1, while no other species were merged in this way. This further supports that both lineages should be considered as the same species (Supplementary Fig. 8d).

Low F_{st} between Clonal and Wild-Type lineages suggests intraspecific differentiation

To evaluate differentiation between the clonal and wild-type lineages, we calculated the F_{st} fixation index, a classical statistic in population genetics that quantifies how much of the total genetic variation is due to differences among groups rather than within them[95]. To avoid under-estimating genetic variation within clonal males due to their haploidy, we ensured an equal number of chromosomes in each population by including twice as many clonal male genomes (n=12, with same individuals than in Fig S14a plus SH19-04, RFOU2M, SH04-20, SH13-14, SH14-07 and SH14-05) compared to wild-type individuals (n=6). F_{st} calculations were performed for each locus individually (n=5,837).

If clonal and wild-type lineages belong to the same species, we expect lower F_{st} values compared to inter-specific pairs. Conversely, we expect similar F_{st} values than in intra-specific pairs. To test this, we repeated the analysis on all inter-specific pairs, using whole-genome data from Supplementary Fig. 8a. For intra-specific comparisons, we divided each species into two populations based on geographical origin and conducted the same analysis.

The results are presented in Supplementary Fig. 9. As expected, F_{st} values for intra-specific pairs are all lower than those for inter-specific pairs. Interestingly, the clonal/wild-type comparison falls within the range of intra-specific pairs. The F_{st} distribution for the clonal/wild-type pair does not significantly differ from that of the eastern and western *M. structor* wild-type pair (two-sided Wilcoxon rank sum test, p-value = 0.7718), while it differs significantly from all other pairs (two-sided Wilcoxon rank sum test, p-value < 2.2×10^{-16}). This indicates that the clonal lineage has not diverged more than two populations of wild-type individuals, suggesting again that they all belong to the *M. structor* species. Interestingly, the lowest F_{st} values were observed within clonal male populations, reflecting the expected low genetic diversity among clones. In contrast, the highest F_{st} values were observed between *M. ibericus* and *M. structor* clonal males. This strongly supports the idea that *M. ibericus* queens produce two distinct species, with genomes that never recombine despite sharing the same nests.

Low genetic divergence between clonal and wild-type lineages falls within intraspecific range

To determine if the genetic difference between clonal and wild-type lineages indicates species-level divergence, we summed branch lengths for each of the 630 pairs of individuals from the phylogenetic tree of Supplementary Fig. 8a. We divided all our pairs into 3 groups, namely intraspecific pairs (both individuals assigned to the same species, n=90), interspecific pairs (individuals assigned to different species, n=504) and Clonal/Wild pairs (one individual is a clonal male while the other is a wild-type *M. structor,* n=36).

Distribution of each pair divergence type is shown in Supplementary Fig. 10a. Divergence between clonal and wild-type individuals, ranging from 0.14% to 0.17%, falls entirely within the range of intraspecific divergences (0.00014% to 0.28%) and does not overlap with interspecific divergence (0.19% to 0.69%). This supports the idea that the genetic divergence between clonal and wild-type lineages is not high enough to justify classifying them as different species.

High historical gene flow between clonal and wild-type lineage does not significantly differ from intraspecific gene flow

To assess whether clonal males and wild-type females are interfertile, we inferred historical gene flow in our dataset. Gene flow refers to the transfer of genetic material across populations, a process occurring primarily through the breeding of individuals between populations. For this, we used *Aphid*, an approximate likelihood approach estimating the proportion of gene tree conflict due to gene flow[96]. This approach analyzes the topologies and branch lengths of triplet gene trees to determine whether conflicts with the species tree are due to gene flow or incomplete lineage sorting, the latter occurring when within-species polymorphism persists longer than the interval between speciation events.

We perform this analysis on every possible triplet of the Supplementary Fig. 8a phylogenetic tree (n=2155), and divided the triplets into 3 groups, namely intraspecific triplets (where the three individuals belong to the same species, n=100), interspecific triplets (where one individual differ from the others, n=1965) and Wild vs Clonal triplets, defined as triplets of two wild-type individuals and one clonal male (n=90).

Distributions of the estimated gene flow proportion (proportion of genes affected by gene flow) of each of the categories are plotted on Supplementary Fig. 10b. Gene flow proportion of clonal/wild-type triplets does not significantly differ from intraspecific triplets (two-sided Wilcoxon rank-sum test, p-value = 0.33), with a nearly equal average of 0.495 and 0.494, respectively. This indicates that the clonal and wild-type lineages have exchanged genes to the same extent as two individuals from the same species, supporting long-lasting interfertility.

Conclusion

The concept of species has several definitions depending on the considered criteria, for example based on morphology (morphological species concept), genetic similarity (phylogenetic species concept) or reproductive isolation (biological species concept)[97,98]. While it is clear that clonal males appear as different from wild-type M. structor according to the morphological species concept (Supplemental Text S3), they appear as part of M. structor according to the phylogenetic species concept (Supplementary Figs. 8-10). The analysis about gene flow (Supplementary Fig. 10b) tests reproductive isolation rather than genetic similarity, which suggests historical interfecundity between clonal and wild-type lineages, in line with the biological species concept.

While clonal males have extensively exchanged genes with the wild-type lineage through their history, they currently never or rarely hybridize with wild-type females (Supplementary Fig. 7). This aligns with limited mating opportunities due to their recent “domesticated condition” within *M. ibericus* nests. As a domesticated lineage, they share the ambiguous taxonomic status of many domesticated species, which also often exhibit divergent phenotypes in spite of high genetic similarity to their wild relatives[99]. As they are produced by different species’ mothers,

domesticated M. structor males are also distinguished by a M. ibericus mitochondria, which may further limit mating with their wild relatives[100].

Input and output files used for the analyses are available in the zenodo repositiry (https://zenodo.org/records/11506545)

Supplementary Figures

Supplementary Fig. 1

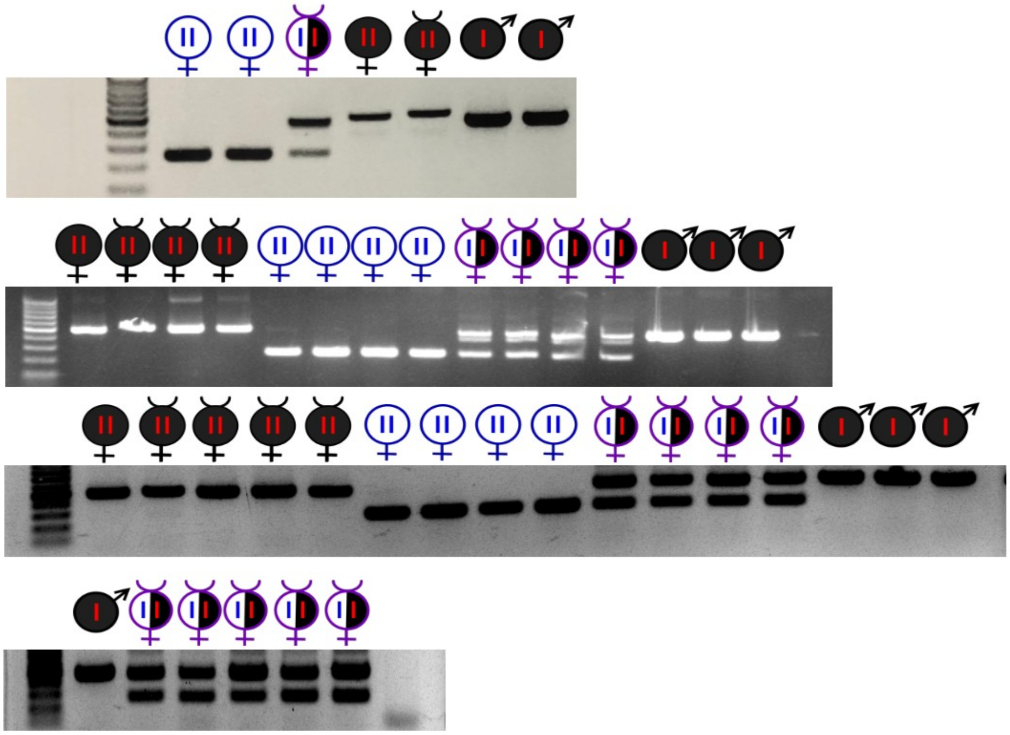

Supplementary Fig. 1: Species and hybrid detection by PCR. Results on agarose gel from 3 PCR runs (3rd and 4th row are from the same run) on adult individuals for validating the approach. Migration goes from top to bottom. Patterns of short, long or short/long fragments respectively match *M. ibericus* (blue female symbols), *M. structor* (red males, females and worker symbols) and hybrid genomes (blue/red worker symbols). For gel source data, see Supplementary Fig. 11.

Supplementary Fig. 2

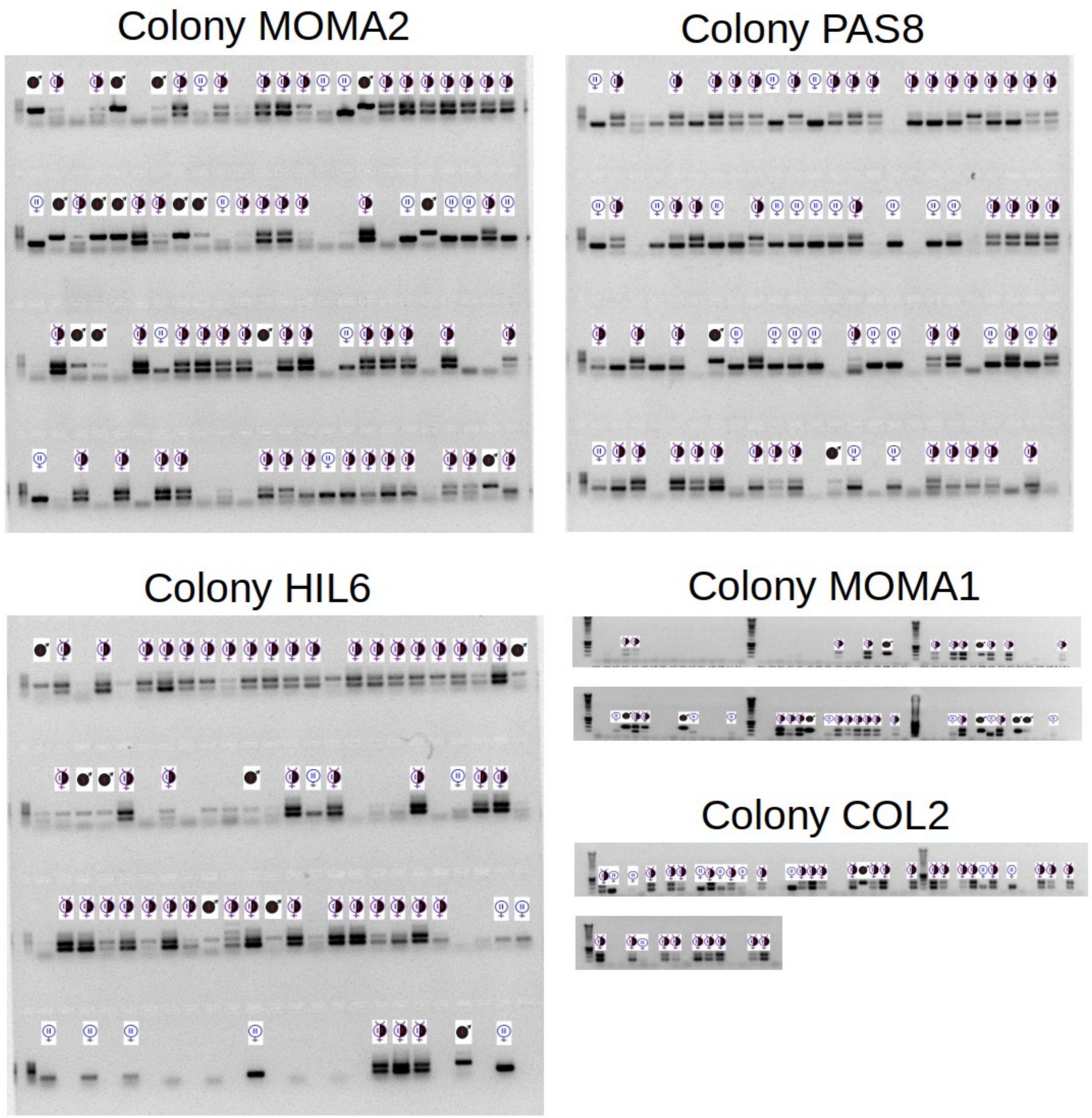

Supplementary Fig. 2: Species identification of *M. ibericus* colonies brood. Results on agarose gel of PCR tests on 5 colonies brood (egg + larvae). Blue female, hybrid worker and red male symbols are indicated when an egg is identified as respectively *M. ibericus* female/male, *M. ibericus/structor* worker or *M. structor* clonal male. When blank, the amplification is considered as ambiguous and is counted as “not genotyped” in Supplementary Table 2. The ID of the considered colony is indicated above each gel. For gel source data, see Supplementary Fig. 11.

Supplementary Fig. 3

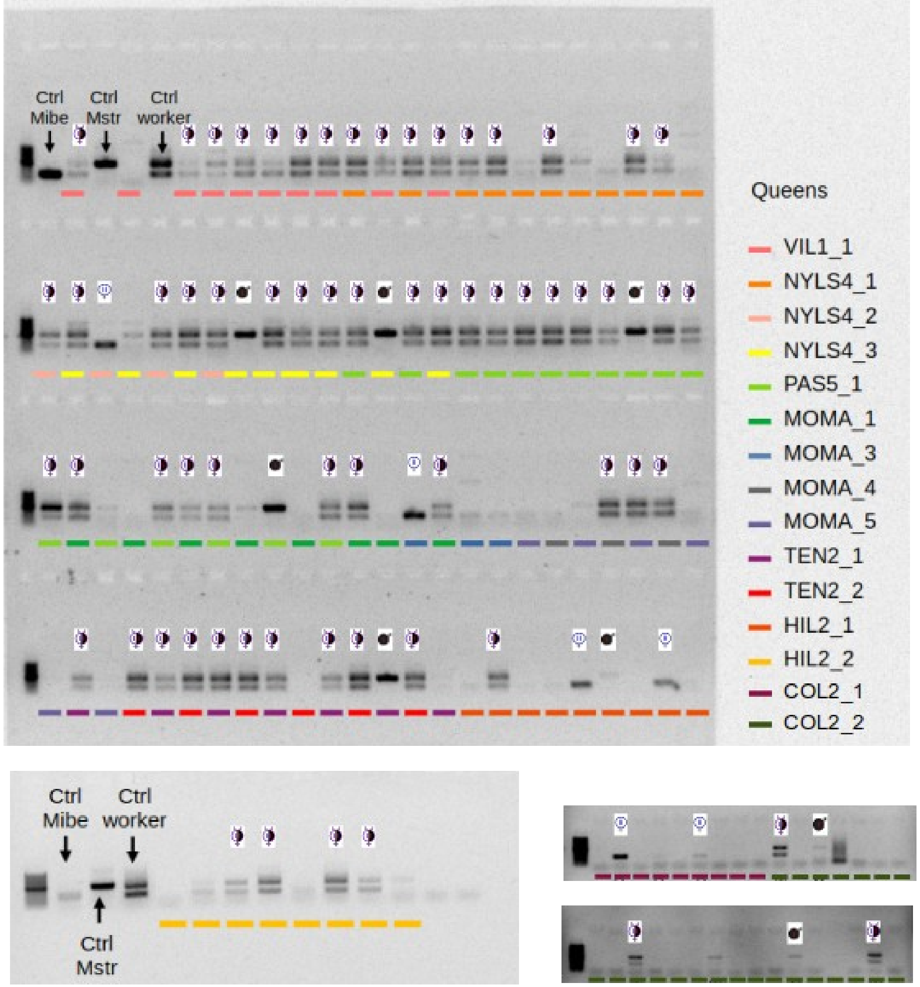

Supplementary Fig. 3: Species identification of eggs from isolated *M. ibericus* queens. Results on agarose gel of PCR tests on 15 queen eggs from 7 different colonies. Blue female, hybrid worker and red male symbols are indicated when an egg is considered identified as respectively *M. ibericus* female/male, *M. ibericus/structor* worker or *M. structor* clonal male. When blank, the amplification is considered as ambiguous and is counted as “not genotyped” in Supplementary Table 3. ID of the considered queens is indicated by a colored dash. For gel source data, see Supplementary Fig. 11.

Supplementary Fig. 4

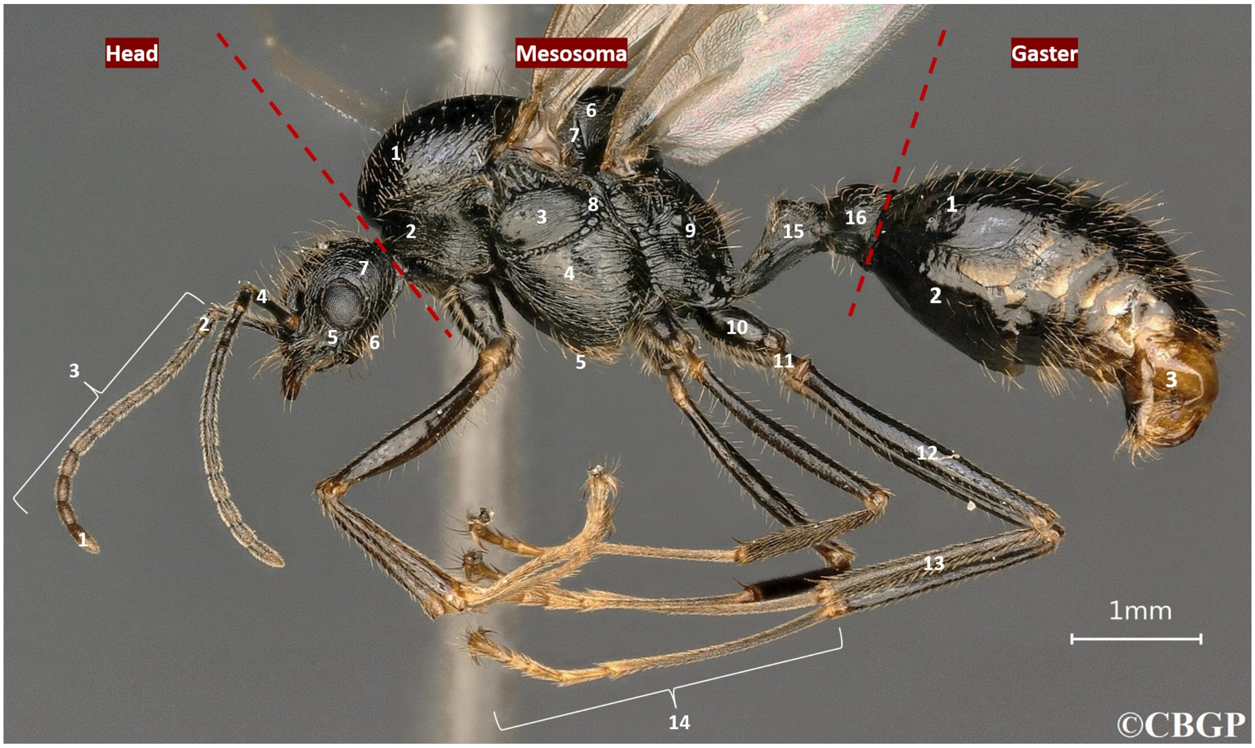

Supplementary Fig. 4: Morphological terms of the *M. structor* (clonal morph) holotype body parts. Head: 1 - Flagellomere; 2 - Pedicel; 3 - Funiculus; 4 - Scapus; 5 - Gena; 6 - Gula; 7 - Temple. Mesosoma: 1 - Mesoscutum; 2 - Pronotum; 3 - Anepisternum; 4 - Katepisternum; 5 - Mesopectum; 6 - Mesoscutellum; 7 - Scutoscutellar sulcus; 8 - Mesopleural sulcus; 9 - Propodeum; 10 - Coxa; 11 - Trochanter; 12 - Femur; 13 - Tibia; 14 - Tarsus; 15 - Petiole; 16 - Postpetiole. Gaster: 1 - First gastral tergite; 2 - First gastral sternite; 3 - Genitalia.

Supplementary Fig. 5

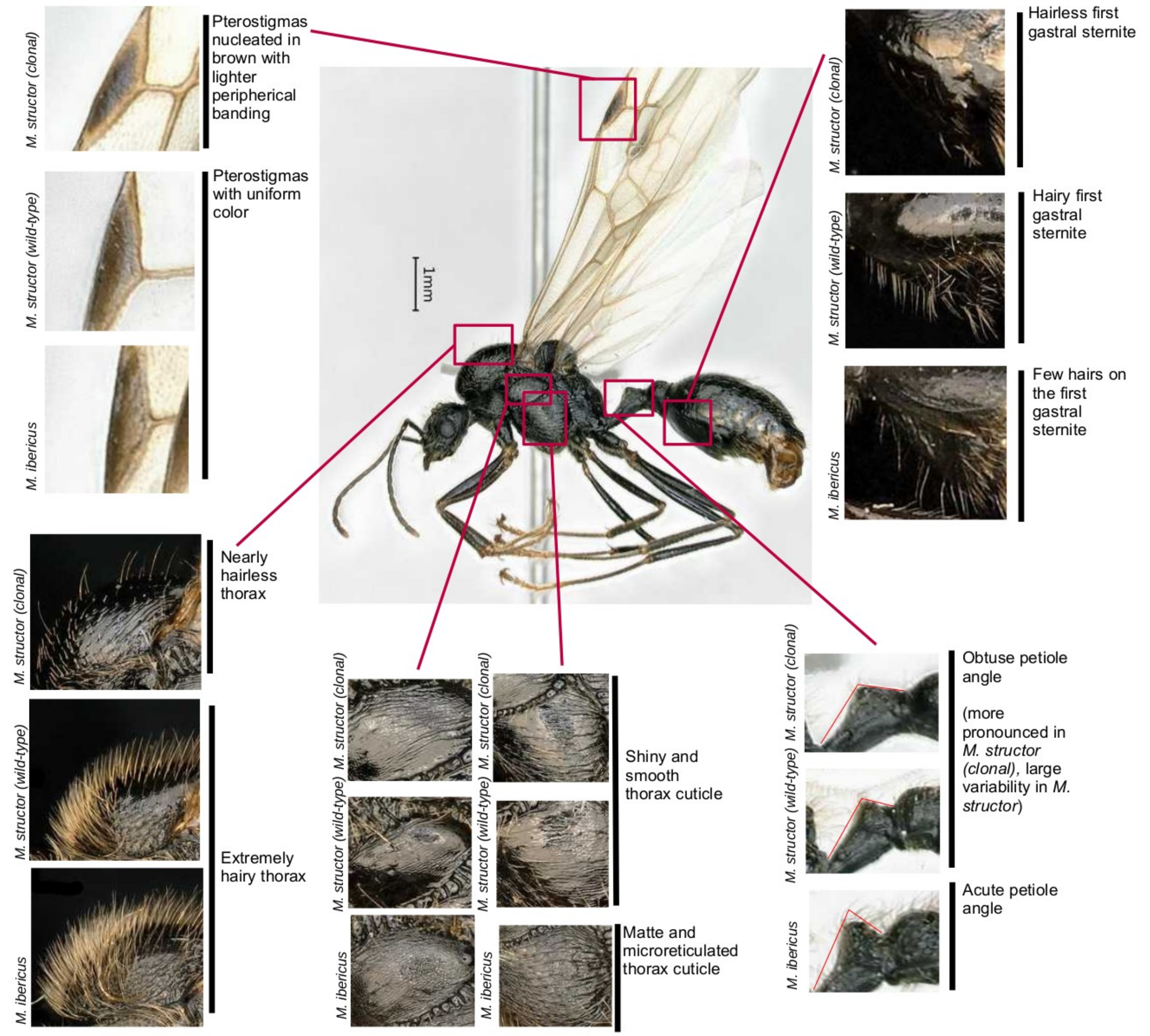

Supplementary Fig. 5: Criteria differentiating *M. structor* males (clonal morph) with *M. structor* (wild-type morph) and *M. ibericus* males. The whole body of the male is the holotype of *M. structor* (clonal morph).

Supplementary Fig. 6

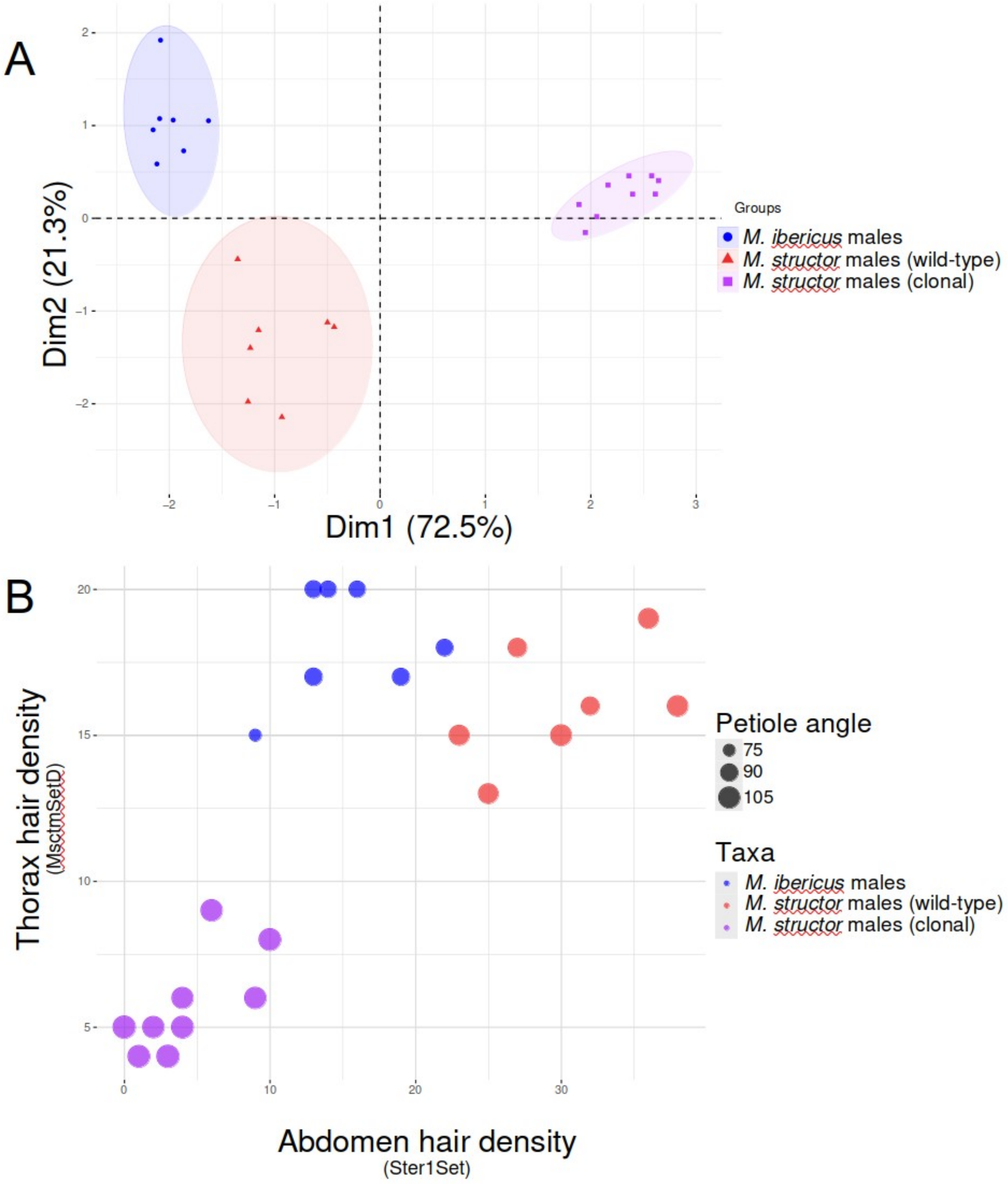

Supplementary Fig. 6: Morphological analyses discriminating males of *M. structor* (wild-type morph; n=7) and *M. ibericus* (n=7) from *M. structor* (clonal morph; n=9). A. PCA with the 5 morphological variables of Supplementary Table 5 discriminate males in 3 groups. **B.** Plot of pilosity density proxies of the thorax (*i.e.* mesosoma) and abdomen (*i.e* gaster) in regards to angle of the abdomen/thorax junction (petiole).

Supplementary Fig. 7

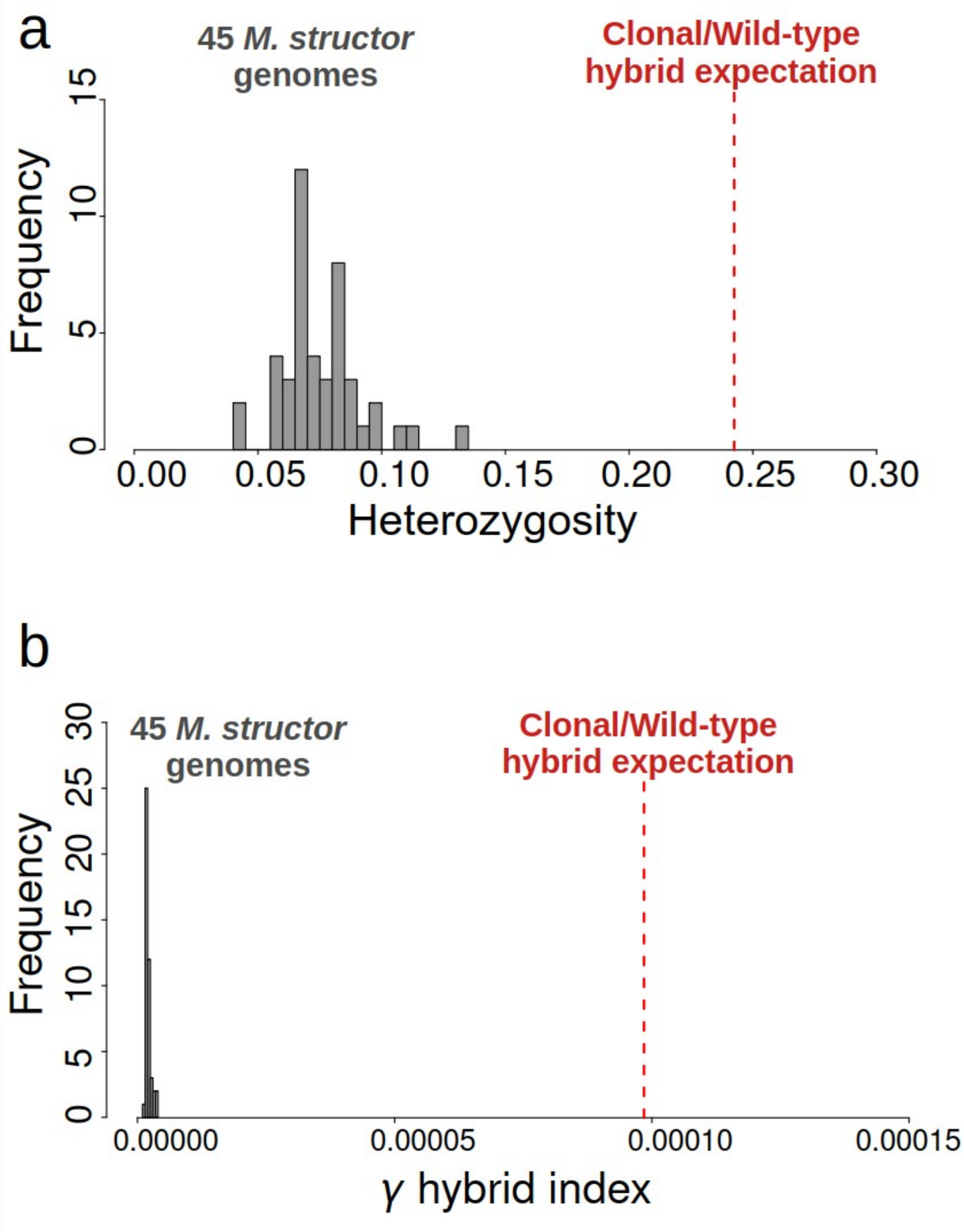

Supplementary Fig. 7: SNP heterozygosity and *γ* **hybrid index distribution of 45** ***M. structor*** **genomes.** The expected value in case of Clonal/Wild-type hybrid is in red and has been inferred from *in silico* hybridization using one haploid male genome from each lineage. **a,** Heterozygosity computed on 5856 loci, average values among 45 *M. structor* genomes significantly differs from the Clonal/Wild-type heterozygosity expectation (two-sided Wilcoxon rank sum test, p-value < 2.2 x 10^{-16}). **b**, *γ* hybrid index computed on 832 loci common to all metazoa, average values among 45 *M. structor* genomes significantly differs from the Clonal/Wild-type *γ* value expectation (two-sided Wilcoxon rank sum test, p-value < 2.2 x 10^{-16}).

Supplementary Fig. 8

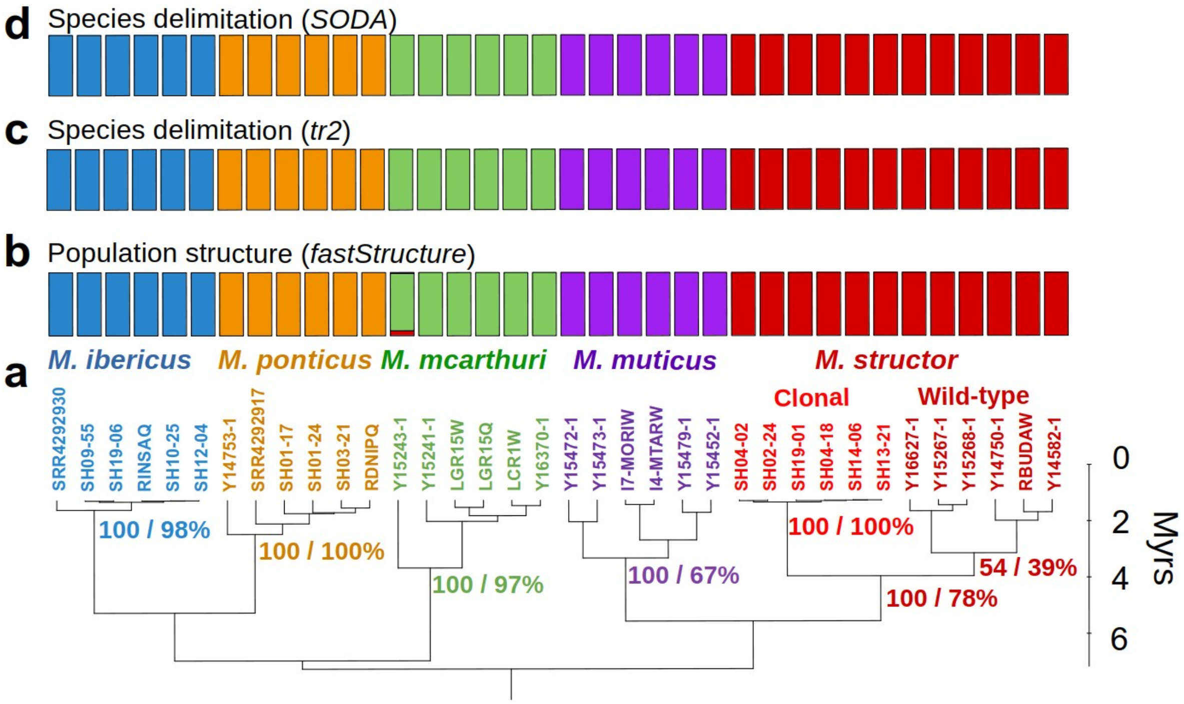

Supplementary Fig. 8: Phylogenetic tree and species delimitation. **a**, Phylogenetic tree of top 6 representative individuals per species (inferred from a 11,102,444 bp supermatrix with IQ-TREE). Support values supporting the monophyly of each species are written in the corresponding color. First number is the ultrafast bootstrap value, second is the Site Concordance Factors (sCF). **b,** Population ancestry proportion computed on each genome (n=5800 loci) from population structure analysis. **c,** Species delimitation based on gene trees (n=5654) using *tr2*. Same color bar refers to the same species assignment by the method. **d**, Species delimitation based on gene trees (n=5804) using *SODA*. Same color bar refers to the same assignment by the method.

Supplementary Fig. 9

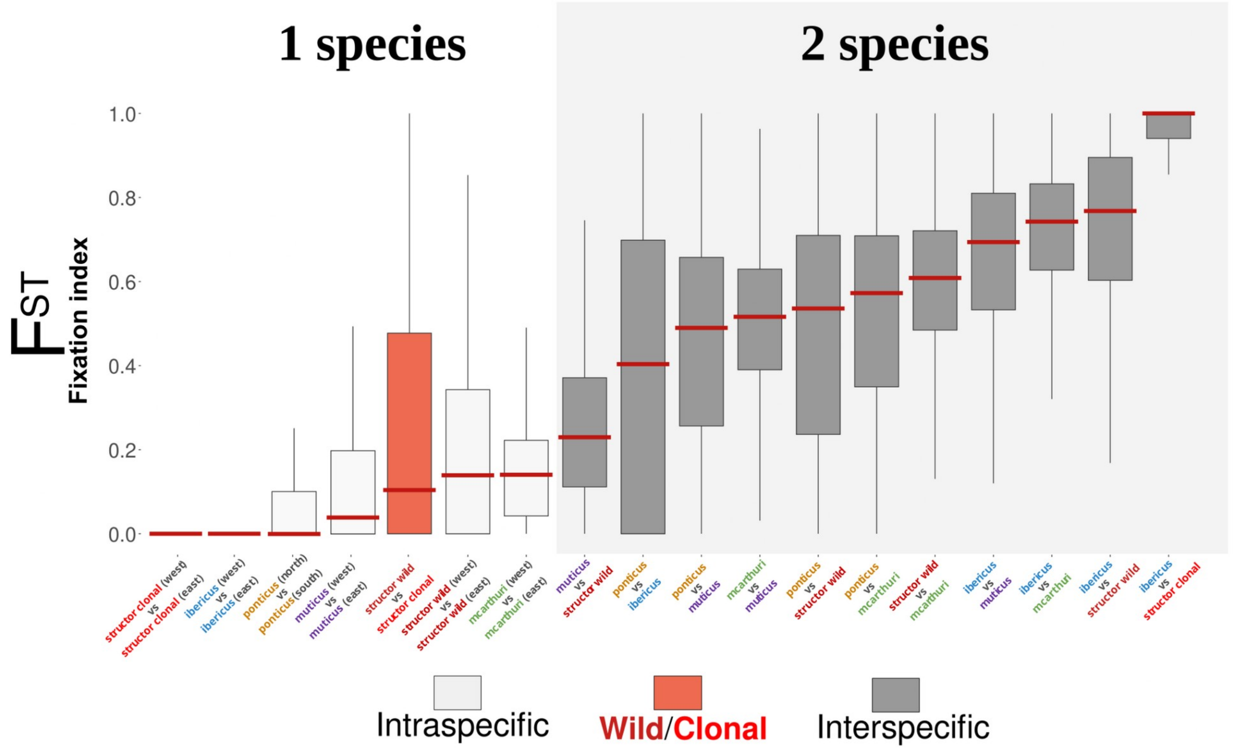

Supplementary Fig. 9: F_{st} Variation across intraspecific and interspecific pairs. Pairs are ranked according to their median F_{st} values (n=5855 loci). Red bars represent medians, boxplot limits represent 1.5x interquartile range. For intraspecific comparisons, we divided each species into two populations based on geographical origin. ***M.structor clonal*** **west** (SH04-18-reseq, SH19-01) vs ***M.structor clonal*** **east** (SH04-02-reseq, SH02-24-reseq); ***M.ibericus*** **west (**SH09–55; SH19-06) vs ***M.ibericus*** **east** (SH10-25, SH12-04); ***M.muticus*** **west** (Y15472-1, Y15473-1) vs ***M.muticus*** **east** (Y15479-1, Y15452-1); ***M.ponticus*** **north** (SH03-2, RDNIPQ) vs ***M.ponticus*** **south** (Y14753-1, SH01-17); ***M.structor wild-type*** **west** (Y16627-1, Y15267-1, Y15268-1) vs ***M.structor wild-type*** **east** (Y14750-1; RBUDAW, Y14582-1); ***M.mcarthuri*** **west** (LGR15Q, Y16370-1) vs ***M.mcarthuri*** **east** (Y15243-1; Y15241-1).

Supplementary Fig. 10

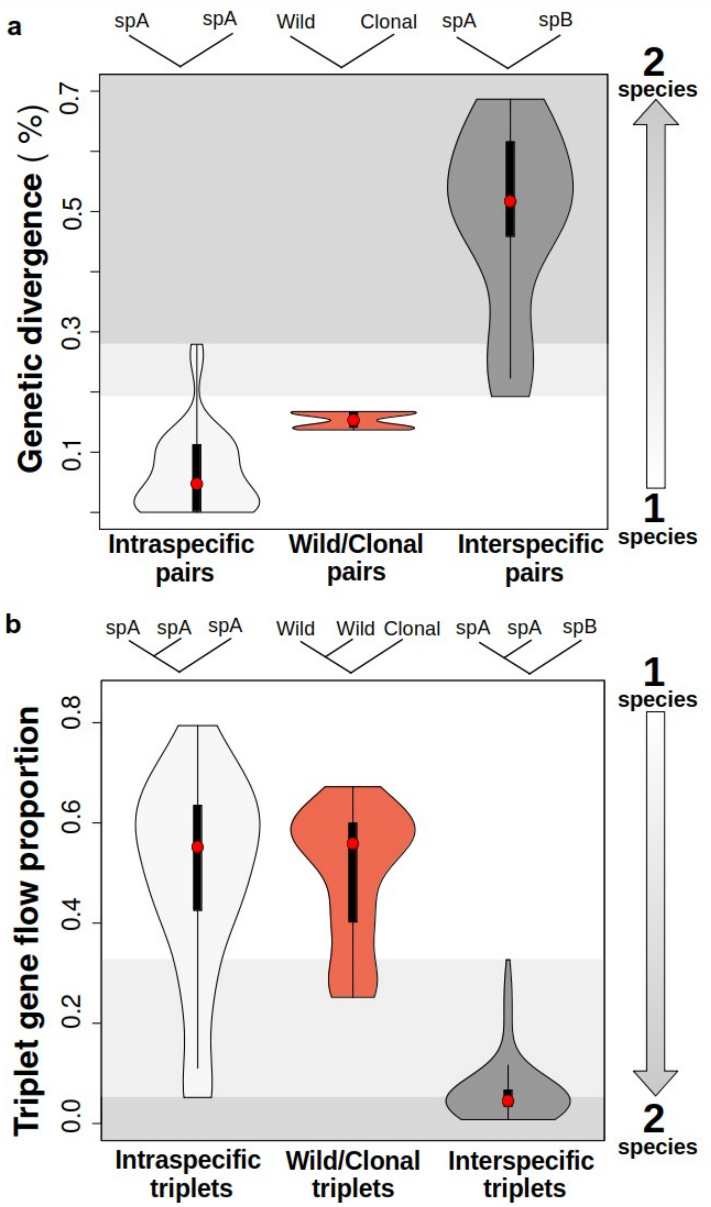

Supplementary Fig. 10: Divergence and gene flow between wild-type and clonal lineage compared with intra- and interspecific comparisons. Red dots represent average values, boxplot limits represent 1.5x interquartile ranges, violin plot delimitaitions represent the total range of the distributions. **a,** Genetic divergence between all wild-type vs. clonal pairs (n=36) compared with intraspecific pairs (n=90) and interspecific pairs (n=504). Dark grey background delimits the divergence range of interspecific pairs, white background the intraspecific one, light grey the divergence range where both overlap. **b**, Gene flow proportion inference within all ((wild,wild),clonal) triplets (n=90) compared with intraspecific triplets (n=100) and interspecific triplets of 2 species (n=1965). Dark grey background delimits the gene flow proportion range of interspecific triplets, white background the intraspecific one, light grey the overlap. Gene flow proportion of clonal/wild-type triplets does not significantly differ from intraspecific triplets (two-sided Wilcoxon rank-sum test, p-value = 0.33)

Supplementary Fig. 11

Used in Supplementary Fig. 1

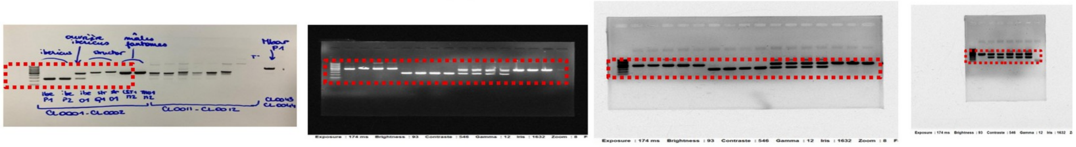

Used in Supplementary Fig. 2

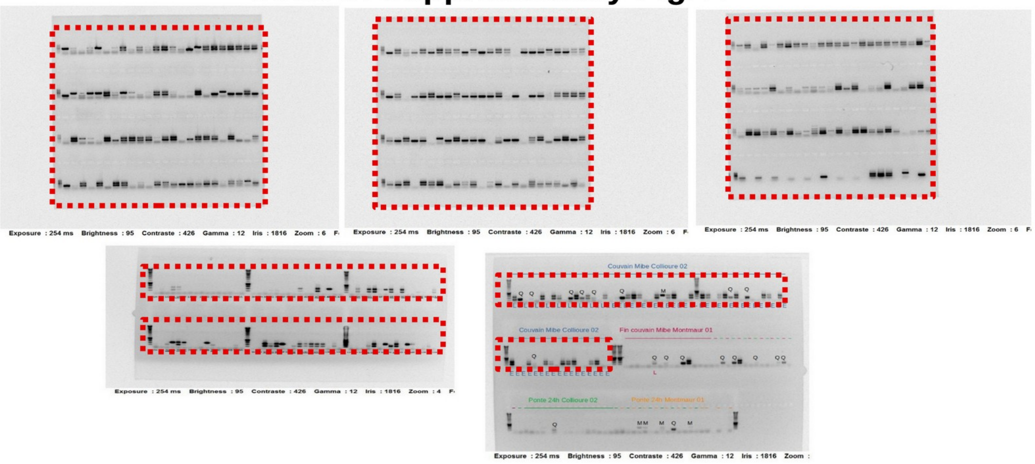

Used in Supplementary Fig. 3

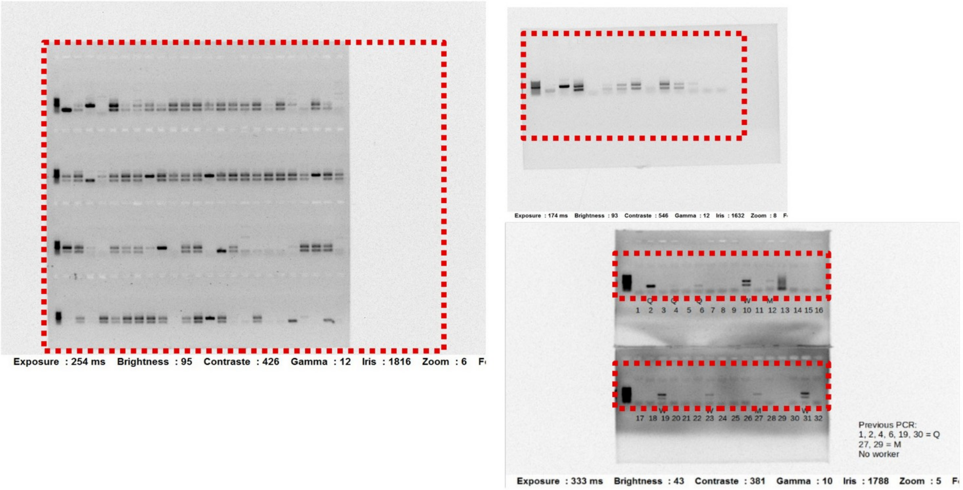

Supplementary Fig. 11: Uncropped gel source data used in Supplemental Figs. 1-3.

Supplementary Tables

Supplementary Table 1

Supplementary Table 1 (provided as separate xls file). Detailed information for each sequenced individual. Column description is included in the xls file in sheet 2.

Supplementary Table 2

Colony	Brood number	*M. ibericus* male/female	*M. structor* clonal males	hybrid worker	Total genotyped	Total not identified
MOMA1	96	7	8	23	38	58
COL2	56	6	1	29	36	20
MOMA2	96	13	14	49	76	20
HIL6	84	9	8	46	63	21
PAS8	96	24	2	47	73	23
Total count	428	59	33	194	286	142
Percent of genotyped brood		**20.629**	**11.538**	**67.832**	**100**	

Supplementary Table 2 : Count and percentage of species identification PCR tests on colony brood (eggs/larvae).

Supplementary Table 3

Colony	Queen ID	Egg number	***M. ibericus*** **male/females**	***M. structor*** **(clonal) males**	**hybrid worker**	**Total genotyped**	**Not identified**
**VIL1**	1	10	0	0	9	9	1
**NYLS4**	1	11	0	0	8	8	3
**NYLS4**	2	4	1	0	3	4	0
**NYLS4**	3	9	0	2	6	8	1
**PAS5**	1	17	0	2	14	16	1
**MOMA2**	1	8	0	0	4	4	4
**MOMA2**	2	0	0	0	0	0	0
**MOMA2**	3	3	1	0	0	1	2
**MOMA2**	4	3	0	0	2	2	1
**MOMA2**	5	6	0	0	1	1	5
**TEN2**	1	7	0	1	5	6	1
**TEN2**	2	6	0	0	5	5	1
**HIL2**	1	9	2	1	1	4	5
**HIL2**	2	8	0	0	4	4	4
**COL2_1**	1	9	2	0	0	2	7
**COL2_2**	2	23	0	1	3	4	19
**TOTAL**		133	6	7	65	78	55
**Percent of genotyped eggs**			**7.692**	**8.974**	**83.333**	**100**	

Supplementary Table 3 : Count and percentage of species identification PCR tests on individual queen eggs.

Supplementary Table 4

	**Number of individuals analyzed**	**Total number of SNPs**	**Synonymous diversity (piS)**	**Non-synonymous over synonymous diversity ratio (piN/piS)**
M. ibericus	**37**	159,886	**0.00045** [0.00043; 0.00048]	**0.162** [0.154; 0.170]
M. ponticus	**7**	54,519	**0.0034** [0.0033; 0.0035]	**0.120** [0.115; 0.126]
M. mcarthuri	**6**	69,030	**0.0056** [0.0055; 0.0057]	**0.121** [0.117; 0.125]
M. muticus	**7**	71,060	**0.0042** [0.0041; 0.0043]	**0.124** [0.119; 0.129]
M. structor (wild-type)	**47**	420,241	**0.0014** [0.0013; 0.0015]	**0.211** [0.203; 0.219]
M. structor (clonal)	**8**	27,147	**0.00027** [0.00021; 0.00033]	**0.427** [0.378; 0.485]

Supplementary Table 4 : Genetic diversity on synonymous sites and non-synonymous over synonymous diversity. Confidence intervals are indicated in brackets.

Supplementary Table 5

Supplementary Table 5 (provided as separate xls file): Details and morphological measurements of males inspected for morphological analyses. Description of morphological criteria is available on sheet 2.

SI References

89. Suni, S. S., Gignoux, C. & Gordon, D. M. Male parentage in dependent-lineage populations of the harvester ant *Pogonomyrmex barbatus*. *Mol. Ecol.* **16**, 5149–5155 (2007).
90. Schwander, T., Keller, L. & Cahan, S. H. Two alternate mechanisms contribute to the persistence of interdependent lineages in *Pogonomyrmex* harvester ants. *Mol. Ecol.* **16**, 3533–3543 (2007).
91. Mo, Y. K., Lanfear, R., Hahn, M. W. & Minh, B. Q. Updated site concordance factors minimize effects of homoplasy and taxon sampling. *Bioinformatics* **39**, (2023).
92. Puechmaille, S. J. The program structure does not reliably recover the correct population structure when sampling is uneven: subsampling and new estimators alleviate the problem. *Mol. Ecol. Resour.* **16**, 608–627 (2016).
93. Fujisawa, T., Aswad, A. & Barraclough, T. G. A rapid and scalable method for multilocus species delimitation using Bayesian model comparison and rooted triplets. *Syst. Biol.* **65**, 759–771 (2016).
94. Rabiee, M. & Mirarab, S. SODA: multi-locus species delimitation using quartet frequencies. *Bioinformatics* **36**, 5623–5631 (2021).
95. Weir, B. S. & Cockerham, C. C. Estimating F-statistics for the analysis of population structure. *Evolution* **38**, 1358 (1984).
96. Galtier, N. An approximate likelihood method reveals ancient gene flow between human, chimpanzee and gorilla. *Peer Community Journal* **4**, (2024).
97. De Queiroz, K. Species concepts and species delimitation. *Syst. Biol.* **56**, 879–886 (2007).
98. Stankowski, S. *et al.* Toward the integration of speciation research. *Evolutionary Journal of the Linnean Society* **3**, (2024).
99. Zeller, U. & Göttert, T. The relations between evolution and domestication reconsidered - Implications for systematics, ecology, and nature conservation. *Global Ecology and*

Conservation **20**, e00756 (2019).

100. Moran, B. M. *et al.* A lethal mitonuclear incompatibility in complex I of natural hybrids. *Nature* **626**, 119–127 (2024).
