# Supplementary material for: One mother for two species via obligate cross-species cloning in ants
Source: Nature. 2025 Sep 3;646(8084):372–7. doi: 10.1038/s41586-025-09425-w (PMC12507663; doi:10.1038/s41586-025-09425-w)
Supplement: Supplementary file 2 — Reporting Summary [file 41586_2025_9425_MOESM2_ESM.pdf]

nature portfolio

Corresponding author(s): Jonathan Romiguier

Last updated by author(s): 28/06/2025

Reporting Summary

Nature Portfolio wishes to improve the reproducibility of the work that we publish. This form provides structure for consistency and transparency in reporting. For further information on Nature Portfolio policies, see our Editorial Policies and the Editorial Policy Checklist.

Statistics

For all statistical analyses, confirm that the following items are present in the figure legend, table legend, main text, or Methods section.

n/a	Confirmed	
☐	☒	The exact sample size (*n*) for each experimental group/condition, given as a discrete number and unit of measurement
☒	☐	A statement on whether measurements were taken from distinct samples or whether the same sample was measured repeatedly
☐	☒	The statistical test(s) used AND whether they are one- or two-sided<br>*Only common tests should be described solely by name; describe more complex techniques in the Methods section.*
☒	☐	A description of all covariates tested
☒	☐	A description of any assumptions or corrections, such as tests of normality and adjustment for multiple comparisons
☐	☒	A full description of the statistical parameters including central tendency (e.g. means) or other basic estimates (e.g. regression coefficient) AND variation (e.g. standard deviation) or associated estimates of uncertainty (e.g. confidence intervals)
☐	☒	For null hypothesis testing, the test statistic (e.g. *F*, *t*, *r*) with confidence intervals, effect sizes, degrees of freedom and *P* value noted<br>*Give P values as exact values whenever suitable.*
☐	☒	For Bayesian analysis, information on the choice of priors and Markov chain Monte Carlo settings
☒	☐	For hierarchical and complex designs, identification of the appropriate level for tests and full reporting of outcomes
☒	☐	Estimates of effect sizes (e.g. Cohen's *d*, Pearson's *r*), indicating how they were calculated

Our web collection on statistics for biologists contains articles on many of the points above.

Software and code

Policy information about availability of computer code

Data collection	No software was used for data collection
Data analysis	Data were analysed on R v. 4.1.2. All other softwares used for analysing genetic data are described in the Material and Methods of the article, including: Wtbg2 v. 2.5, MaSurCA v. 3.4.1, NextPolish v. 1.3.1, RagTag v. 1.0.2, TGS-GapCloser v. 1.1.1, QUAST v. 5.0, BUSCO v. 4.0.5, fastp v. 0.23.2, BWA-MEM2 v. 2.2.1, GATK v. 4.3, vcftool v. 0.1.16, MAFFT v. 7.490, trimal v. 1.4, IQ-TREE v. 2.07/v. 2.12/v. 2.2.2.7, PAML v. 4.10.7, Tracer v. 1.7.2, PLINK v. 1.90b6.21, fastStructure v. 1.0 (includes chooseK.py), dNdSpiNpiS v. 1.0, tr2 v. 1, SODA v. 1.0.2, aphid v. 0.11. Scripts used for producing the results of the study are available in the Zenodo repository at the following URL: https://zenodo.org/records/11506545

For manuscripts utilizing custom algorithms or software that are central to the research but not yet described in published literature, software must be made available to editors and reviewers. We strongly encourage code deposition in a community repository (e.g. GitHub). See the Nature Portfolio guidelines for submitting code & software for further information.

Data

Policy information about availability of data

All manuscripts must include a data availability statement. This statement should provide the following information, where applicable:

- Accession codes, unique identifiers, or web links for publicly available datasets
- A description of any restrictions on data availability
- For clinical datasets or third party data, please ensure that the statement adheres to our policy

Raw reads of genetic data are deposited on NCBI under the Project ID PRJNA1145159 (available with publication). SRA IDs and all data supporting the results of the study are indicated for each sample in Table S1. Reference genomes, genetic variation data, phylogenetic analyses and scripts used for producing the results of the study are available in the zenodo repository at the following URL: https://zenodo.org/records/11506545. We also used the following dataset from the orthoDB database (https://www.orthodb.org/): hymenoptera_odb10 (https://busco-data.ezlab.org/v5/data/lineages/hymenoptera_odb10.2024-01-08.tar.gz) and metazoa_odb10 (https://busco-data.ezlab.org/v5/data/lineages/metazoa_odb10.2024-01-08.tar.gz).

Research involving human participants, their data, or biological material

Policy information about studies with human participants or human data. See also policy information about sex, gender (identity/presentation), and sexual orientation and race, ethnicity and racism.

Reporting on sex and gender	N/A
Reporting on race, ethnicity, or other socially relevant groupings	N/A
Population characteristics	N/A
Recruitment	N/A
Ethics oversight	N/A

Note that full information on the approval of the study protocol must also be provided in the manuscript.

Field-specific reporting

Please select the one below that is the best fit for your research. If you are not sure, read the appropriate sections before making your selection.

☐ Life sciences ☐ Behavioural & social sciences ☒ Ecological, evolutionary & environmental sciences

For a reference copy of the document with all sections, see nature.com/documents/nr-reporting-summary-flat.pdf

Ecological, evolutionary & environmental sciences study design

All studies must disclose on these points even when the disclosure is negative.

Study description	DNA was extracted from ant individuals. Shotgun sequencing data were generated and aligned to a reference genome of Messor ibericus to analyse it.
Research sample	390 ant individuals were analysed in this study, from which 326 whole genomes and 52 transcriptomes have been newly sequenced. These samples range across Europe (from Spain to Turkey). Details are listed in Table S1.
Sampling strategy	Genome-wide data for 390 individuals is considered as above average for population genetics. We focused particularly the sampling on the two species of interest (289 Messor ibericus and 77 Messor structor).
Data collection	Genomic data was collected according to laboratory protocols to minimise contamination (details in Methods Section). DNA and RNA libraries were sequenced via Illumina sequencing.
Timing and spatial scale	Samples range across Europe and were collected between 2000 and 2022.
Data exclusions	No data were excluded.
Reproducibility	Genome and transcriptome sequencing has been repeated several times and all necessary informations are available in the Methods Section. Egg genotyping has also been repeated several time successfully and the Methods Section provide all necessary information.
Randomization	Randomization was not applicable in this study. Samples were grouped into their species origin identified morphologically then genetically.

Blinding | DNA/RNA extraction and library preparations have been performed without initially knowing the species of origin.

Did the study involve field work? ☒ Yes ☐ No

Field work, collection and transport

Field conditions | Performed in spring, summer and autumn across Europe, weather usually sunny, often after a rainfall to maximize the probability to find reproductive individuals.

Location | All locations are described in Table S1.

Access & import/export | All field samples were collected on public property or on private property with permission of the land owner. None of the species collected were endangered or collected on protected lands.

Disturbance | Not applicable.

Reporting for specific materials, systems and methods

We require information from authors about some types of materials, experimental systems and methods used in many studies. Here, indicate whether each material, system or method listed is relevant to your study. If you are not sure if a list item applies to your research, read the appropriate section before selecting a response.

Materials & experimental systems

n/a	Involved in the study	
☒	☐	Antibodies
☒	☐	Eukaryotic cell lines
☒	☐	Palaeontology and archaeology
☐	☒	Animals and other organisms
☒	☐	Clinical data
☒	☐	Dual use research of concern
☒	☐	Plants

Methods

n/a	Involved in the study	
☒	☐	ChIP-seq
☒	☐	Flow cytometry
☒	☐	MRI-based neuroimaging

Animals and other research organisms

Policy information about studies involving animals; ARRIVE guidelines recommended for reporting animal research, and Sex and Gender in Research

Laboratory animals | This study did not involve laboratory animals.

Wild animals | Individuals of various species (M. ibericus, M. structor, M. ponticus, M. mcarthuri and M. muticus), caste (queens, males, workers) and ages (<1 year for males and isolated queens, >1 year for mature colonies) were captured in their natural habitat with tweezers and transported in plastic boxes. Some colonies with queens were maintained in artificial nests in the lab and were monitored for up to 36 months. Other individuals were stored in eppendorf filled with EtOH for conserving their DNA before sequencing their genome. No individuals were released.

Reporting on sex | Sex was identified morphologically and is reported in Supplementary Table 1.

Field-collected samples | Collected ants were reared in fluoned boxes (queens, workers, males) in a room at 28°C with 40% humidity (no light/photoperiod to mimick their underground lifestyle). Nests were cleaned twice a week and they were provided with water and grass seeds. Colonies are either still maintained in the lab or have been maintained until their natural death.

None of the species collected are endangered as determined by their absence on the IUCN Red List of Threatened Species.

Ethics oversight | No ethical approval is required for research on non-endangered invertebrates.

Note that full information on the approval of the study protocol must also be provided in the manuscript.

Plants

Seed stocks	N/A
Novel plant genotypes	N//A
Authentication	N/A
